# Current therapy option for necrotizing enterocolitis: Practicalities and challenge

**DOI:** 10.3389/fped.2022.954735

**Published:** 2022-07-28

**Authors:** Huihuan Wu, Kehang Guo, Zewei Zhuo, Ruijie Zeng, Yujun Luo, Qi Yang, Jingwei Li, Rui Jiang, Zena Huang, Weihong Sha, Hao Chen

**Affiliations:** ^1^Department of Gastroenterology, Guangdong Provincial People’s Hospital, Guangdong Academy of Medical Sciences, Guangzhou, China; ^2^School of Medicine, South China University of Technology, Guangzhou, China; ^3^School of Bioscience and Bioengineering, South China University of Technology, Guangzhou, China; ^4^Shantou University Medical College, Shantou, China; ^5^Department of General Medicine, Guangdong Provincial People’s Hospital, Guangdong Academy of Medical Sciences, Guangzhou, China

**Keywords:** necrotizing enterocolitis, breast milk composition, stem cell, fecal microbiota transplantation, immunotherapy

## Abstract

Necrotizing enterocolitis (NEC) is one of the most prevalent neonatal gastrointestinal disorders. Despite ongoing breakthroughs in its treatment and prevention, the incidence and mortality associated with NEC remain high. New therapeutic approaches, such as breast milk composition administration, stem cell therapy, immunotherapy, and fecal microbiota transplantation (FMT) have recently evolved the prevention and the treatment of NEC. This study investigated the most recent advances in NEC therapeutic approaches and discussed their applicability to bring new insight to NEC treatment.

## Introduction

Necrotizing enterocolitis (NEC) is an inflammatory bowel disease that is particularly dangerous in premature or low-birth-weight babies (1). Despite tremendous advancements in NEC treatment and neonatal care over the past few decades, the current state of treatment remains unsatisfactory, and mortality and morbidity remain high (2). Short bowel syndrome and intestinal failure are possible outcomes of surgical resection of the necrotic part of the intestine. Patients who survive NEC have a higher risk of developing long-term complications, such as neurodevelopmental delay (3, 4).

**FIGURE 1:**
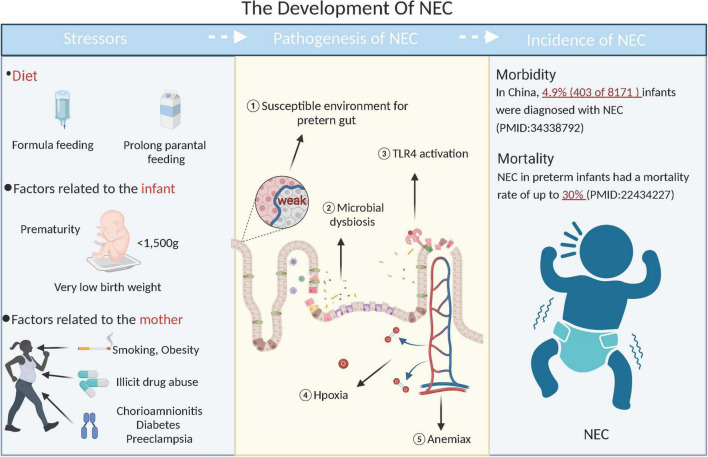
The mechanisms of NEC. It is thought that NEC has complex and multiple mechanisms.

Prevalence and development of NEC are extraordinarily complex. Low birth weight, prolonged parenteral feedings, and short gestation periods are all risk factors of preterm birth. Additionally, mother’s lifestyle (such as smoking and obesity), the prevalence of associated disorders (such as diabetes mellitus, preeclampsia, and chorioamnionitis), and prenatal medications (such as antibiotics and corticosteroids) are risk factors for NEC (Figure 1) (5–10). There are multiple factors involved in developing NEC, including genetic susceptibility, immature intestinal host defense, abnormal microbiota colonization, hypoxia, ischemia, hyperresponsiveness of the intestinal mucosa (11, 12). Despite the study of NEC from various angles, the mechanisms that cause the disease are still largely unknown, which impedes its development into a specific treatment. Prevailing treatment strategies for NEC include antibiotics, surgery, and advanced life support, but their effect is limited. Therefore, a more effective approach to treating NEC is necessary.

**FIGURE 2:**
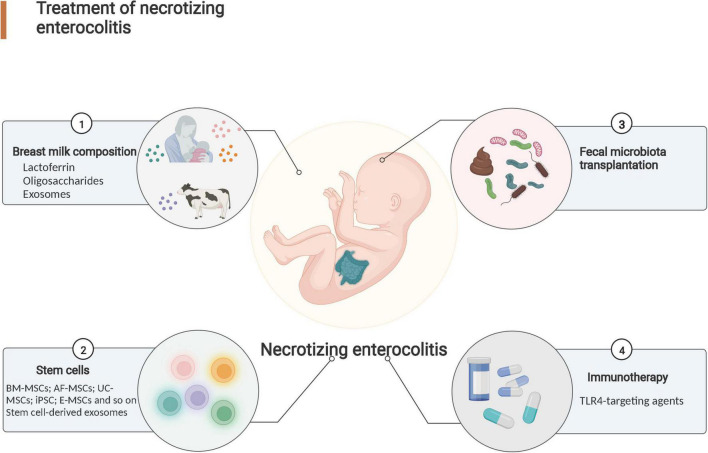
Research progress in necrotizing enterocolitis treatment. Effective treatments for NEC include breast milk composition administration, stem cell therapy, fecal microbiota transplantation (FMT) and immunotherapy.

In this review, breast milk composition, stem cells, immunotherapy, and fecal microbiota transplantation (FMT) were considered the most recent developments in NEC treatment (Figure 2). In addition, their applications to the NEC treatment were evaluated to illuminate the limitations and challenge of the NEC treatment.

## Necrotizing enterocolitis treatment strategy

### Therapy with breast milk composition

Multiple studies have demonstrated that Breast milk composition, including lactoferrin, oligosaccharides, breast milk-derived exosome and so on, is one of the most effective methods for preventing and treating NEC (13, 14). In this section, We focused mainly on lactoferrin, oligosaccharides and breast milk-derived exosome based on their potential applications in NEC prevention and treatment (Table 1).

**TABLE 1:** Applications of breast milk components in NEC.

Molecules in breast milk	Species	Outcomes	Year	References
Lactoferrin	Preterm infants	Reduces IL-6 and TNF-α expression, and upregulates Lgr5^+^ stem cell expression and epithelial proliferation.	2020	(14)
Lactoferrin	Low birth weight neonate	Decrease in IL-10 levels.	2020	(17)
Lactoferrin	Pig	Moderate doses (0.1–1 g/L) enhance cell proliferation and downregulate apoptosis and inflammation. High doses (10 g/L) trigger inflammation.	2016	(16)
Lactoferrin	Very low birth weight neonates	Reduces the incidence and death of > > stage 2 NEC.	2014	(18)
Lactoferrin	Preterm Infant	Reduces the incidence of NEC.	2020	(19)
Oligosaccharides	Mouse	HMOs, accelerate the turnover of crypt cells to protect intestinal epithelial cells from injury.	2019	(23)
DSLNT	Preterm infant	lowers NEC risk.	2018	(21)
Sialylated oligosaccharides	Rat	SHMOs reduce intestinal inflammation by inhibiting TLR4/NLRP3 pathway.	2021	(22)
Oligosaccharide	Pig	HMOs, reduce bowel inflammation.	2017	(24)
HM-EX	Cell	Protected IEC-6 from an oxidative stress injury	2018	(26)
HM-EX	Rat	Protected villous integrity, restored enterocyte proliferation, and improved intestinal epithelial cells	2019	(27)
HM-EX	/	Protected ISCs from oxidative stress injury	2020	(28)
BOVM-EX	Mouse	Improved goblet cell activity, prevented the development of NEC	2019	(32)
RAM-EX	Cell	Promoted IEC viability, enhanced proliferation, and stimulated intestinal stem cell activity	2017	(30)
PM-Ex	Mouse	Decreased intestinal epithelial apoptosis by inhibiting TLR4/NF-κB signaling	2019	(31)

### Lactoferrin

Lactoferrin is the most abundant protein in colostrum (5–6.7 g/L) and is the most important protein found in breast milk (15). Lactoferrin has been demonstrated to inhibit the release of pro-inflammatory cytokines, such as IL-6 and TNF- α, thus reducing intestinal inflammation (14). In addition to maintaining the barrier function of the gut, lactoferrin influences intestinal epithelial cell proliferation and apoptosis (16). Lactoferrin’s effectiveness in preventing and treating NEC has been demonstrated in several preclinical studies and clinical trials (17, 18). Up to now, a phase III clinical trial (ClinicalTrials.gov Identifier: NCT03431558) is currently underway to determine the health effects of lactoferrin with gradient concentration in neonates with low birth weight at the Aga Khan University Hospital, Pakistan. However, based on a systematic review and meta-analysis of nine RCTs with 3515 samples, enteral lactoferrin supplementation did not reduce late-onset sepsis incidences in NEC, all-cause mortality, sepsis-related mortality, NEC stage II or III, and other adverse outcomes (19).

### Oligosaccharides

The significance of Oligosaccharides in protecting against NEC has been a developing area of research since human breast milk is a recognized protective mechanism against the development of NEC. Animals fed a formula containing DSLNT displayed a decrease in NEC severity and lower mortality in preclinical experiments using a newborn rat model of NEC (20, 21). The same study also showed sialylated oligosaccharides, similar to HMOs, but structurally different, decreased NEC incidence and pathological damage scores in rats (22). A rat model of NEC showed that supplementation with sialylated oligosaccharides reduced NEC incidence and intestinal pathology with inhibiting toll like receptor 4/NLRP3 inflammasome pathway (22).

According to a study, oligosaccharides protect intestinal epithelial cells from damage by inhibiting TLR4 expression and increasing crypt cell turnover (23). However, a model of NEC in preterm piglets receiving complex microbial blends did not show any difference in intestinal microbial diversity or protection against NEC (24).

### Breast milk-derived products

**FIGURE 3:**
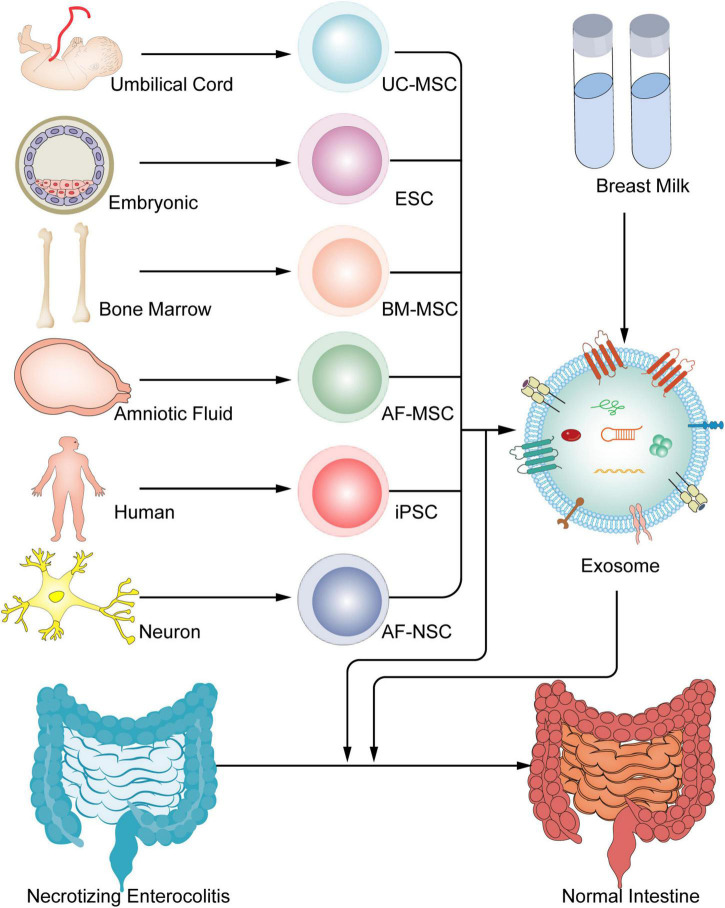
Stem cells and exosomes have been used in the therapy of experimental NECs. Umbilical cord, embryonic, bone marrow, amniotic fluid and so on, have the ability to create stem cells, and all stem cells have the able to generate exosomes, which both can all be treated to treat NEC.

Exosomes are known to include bioactive constituents such mRNA, miRNA, DNA, and proteins and to be produced by a variety of cell types (25). Breast milk-derived exosomes enhance the development of gut and exerts positive impacts on experimental NEC (Figure 3) (26–29). Breast milk-derived exosomes prevent intestinal stem cells from oxidative stress, which were regulated by the Wnt/-catenin signaling pathway (28). In addition, rat milk-derived exosomes increase intestinal stem cell activity, promote IEC viability, and boost proliferation (30). Porcine milk-derived exosomes were reported to protect the intestinal epithelium against LPS-induced injury by inhibiting excessive inflammation and preventing apoptosis through the action of exosome miRNAs (31). Exosomes isolated from bovine milk were administered to protect experimental NEC-induced bowel injury by enhancing goblet cell production and endoplasmic reticulum function (32). According to these studies, breast milk-derived exosomes may exert potential protective effects against NEC.

### Challenge and limitation of breast milk composition therapy

Lactoferrin, oligosaccharides and exosomes in breast milk have protective effects on NEC. However, these are difficult to implement in the clinic. For instance, lactoferrin and oligosaccharides from breast milk have outstanding anti-inflammatory properties, and relevant clinical research is now underway. Still, they are underutilized in clinical settings, making their promotion difficult. To establish the efficacy and long-term benefits of lactoferrin and oligosaccharides and the optimal dose and administration method, higher-quality, well-designed, larger, multicenter clinical trials are required. Furthermore, breast milk-derived exosomes research for the treatment of NEC is still in its early stages. For further verification and in-depth exploration of the mechanism of exosomes in the treatment of NEC, a large number of animal experiments are required.

### Stem cells therapy

Stem cell therapy is increasingly being proposed as a novel therapeutic approach for a variety of diseases, such as spinal cord injury (SCI), stroke (33, 34). Preclinical research on the potential therapeutic role of stem cells in experimental NEC is growing. This section will cover the therapeutic effectiveness of stem cell and stem cell-derived products in the treatment of NEC and provide an overview of ongoing preclinical research (Table 2).

**TABLE 2:** Applications of stem cells and stem cells-derived products in NEC.

Stem cells and stem cells-derived products	Administration	Species	Outcome	Year	References
BM-MSC	Intraperitoneal injection	Rat	Showed weight gains, improve clinical sickness scores, reduced histopathological damage	2011	(35)
BM-MSC	Intraperitoneal injection	Mouse	HB-EGF promoted BM-MSC proliferation, and migration and decreased BM-MSC apoptosis. HB-EGF and BM-MSC act synergistically to reduce injury and improve survival in NEC	2012	(37)
BM-MSC	Intraperitoneal injection Intravenous injection	Rat	Reduced the incidence and severity of NEC, and preserved intestinal barrier function in NEC.	2019	(42)
AF-MSC	Intraperitoneal injection Intravenous injection	Rat	Reduced the incidence, and severity, and preserved intestinal barrier function in NEC.	2019	(42)
AF-MSC	Intraperitoneal injection	Rat	Improved gut barrier function in NEC. AF-MSC, BM-MSC, AF-NSC, and E-NSC all reduce the incidence of NEC, which is not largely different.	2017	(41)
AF-MSC	Intraperitoneal injection	Mice	Rescued intestinal injury and restored epithelial regeneration., increased ISC and epithelial proliferation by Wnt signaling.	2020	(43)
UC-MSC	Intraperitoneal injection	Rat	Improved clinical sickness scores.	2019	(45)
UC-MSC	Intravenous injection	Infant	Enhanced intestinal blood supply in subsequent jejunostomies	2019	(48)
AF-NSC	Intraperitoneal injection Intravenous injection	Rat	Reduced the incidence, and severity, and preserved intestinal barrier function in NEC.	2019	(42)
AF-NSC	intraperitoneal injection	Rat	Reduced the incidence and severity of NEC.	2017	(41)
E-NSC	Intraperitoneal injection	Rat	Reduce the incidence and severity of NEC.	2017	(41)
BM-MSC-Ex	Intraperitoneal injection	Rat	Decreases the incidence and severity of NEC.	2018; 2016	(36, 46)
AF-MSC-EX	Intraperitoneal injection	Mice	Rescued intestinal injury, restored epithelial regeneration, increased ISC and epithelial proliferation by Wnt signaling and decreases the incidence and severity of NEC	2018; 2020	(36, 43)
AF-NSC-EX	Intraperitoneal injection	Rat	Decreases the incidence and severity of NEC.	2018	(36)
E-NSC-EX	Intraperitoneal injection	Rat	Decreases the incidence and severity of NEC.	2018	(36)

### Bone marrow-derived mesenchymal stem cells (BM-MSCs)

In 2011, MSCs were administered intraperitoneally for the first time to treat NEC in rat models. The results illustrate that MSCs could represent a new treatment option for repairing and regenerating injured intestinal tissue in NEC due to their beneficial effects on reducing inflammation and improving tissue regeneration (35). The same study found that intraperitoneal administration of MSCs reduces injury and improves survival in experimental NEC (36). Researchers compared the therapeutic effects of intraperitoneal- and intravenous -administered MSC when treating experimental NEC. They found that intravenous -administered MSC had dramatically improved intestinal engraftment, intravenous administration may be a more effective delivery method than intraperitoneal administration (37). Even though both routes of administration may be used clinically, intravenous administration is a quick and easy way to inject MSC into the body.

### Amniotic fluid-derived mesenchymal stem cells (AF-MSCs)

Amniotic fluid-derived mesenchymal stem cells are cultured using amniotic fluid collected via amniocentesis or cesarean section (38, 39). AF-MSCs therapy has three obvious benefits: AF-MSCs are abundant, are simple to collect and with ease to culture *in vitro* with modest amounts of medium supplement, and develop quicker than BM-MSCs (40). Due to these advantages, AF-MSCs therapy appears to be the optimum stem cell therapy for treating NEC and has piqued the interest of researchers. Other studies also demonstrated that intraperitoneal injection of AF-MSCs decreased the incidence of NEC and enhanced the intestinal barrier function in rats (41, 42). Similarly, Li et al. (43) discovered that Wnt-β signaling increased cell proliferation while decreased inflammatory factor release, restoring intestinal epithelial regeneration after intraperitoneal injection of AF-MSCs.

### Stem cells of other sources

Other sources of stem cells, such as embryonic stem cells (ESCs), umbilical cord-derived mesenchymal stem cells (UC-MSCs), enteral neural stem cells (E-NSCs), amniotic fluid-derived neural stem cells (AF-NSCs) and induced pluripotent stem cells (iPSCs) also have been shown to reduce the incidence of NEC (44, 45). Overall, these findings suggests that stem cell therapy represent a promising treatment for NEC.

### Stem cell-derived products

Exosome may reduce the incidence and severity of experimental NEC as effectively as the stem cells from which they derive (Figure 3) (36). According to the study, they showed that the effect on intestinal injury repair was similar with that of BM-MSCs, AF-MSCs, AF-NSCs, and E-NSCs therapy in rat model of NEC (36). Exosomes produced by AF-MSCs largely activated the Wnt/catenin signaling pathway to increase enterocyte proliferation, reduce inflammatory response, and promote normal intestinal epithelium regeneration (43). Researchers reveal that intraperitoneal -administered BM-MSCs-derived exosomes can independently maintain the integrity of the intestinal barrier from experimental NEC (46). Further, the results of the first comprehensive review and meta-analysis of preclinical models examining the role of stem cells- derived exosomes in experimental NEC demonstrated that exosomes derived from stem cells improved survival and reduced the incidence and severity of cases were diagnosed NEC in rat model (47). The results of these studies suggest exosomes are an effective approach in prevention of NEC development.

### Challenge and limitation of stem cell therapy

Despite these positive outcomes in animal models, there is currently no ongoing stem cell therapy clinical trial for human NEC. Although an instance of supraventricular tachycardia led to a case of NEC. UC-MSCs were administered intravenously to show enhanced intestinal blood supply in subsequent jejunostomies, without any signs of small bowel syndrome (48). A single instance, though, is insufficient to show that stem cell therapy is available in clinics, and there may be other unidentified aspects that merit research as well. Besides, stem cell therapy is limited in the clinical treatment of NEC due to ethical concerns, immunological rejection and a significant risk of tumorigenesis (49–51). Stem cell therapy is a hard task to convert for preclinical and clinical application since it must also address issues including an augmented immune response, cancer, gene mutation, and ethical concerns. It is crucial to find an efficient therapeutic method that does not directly use stem cells in these conditions. Exosome may reduce the incidence and severity of experimental NEC as effectively as the stem cells from which they derive. The use of stem cell-derived exosomes, may be the best way to overcome some of the limitations of stem cell therapy (36).

Exosome therapy is easier to be administered than stem cell therapy because there is no chance of teratoma formation or ethical concerns. However, researchers continue to face considerable challenges in expanding the use of exosome treatment in clinics. Limitations and Challenge might be from three aspects: (1) long-term exosome extraction, low purity, and partial disintegration of obtained exosome (52–56); (2) poor targeting capability and probable “dilution effect” that could reduce treatment efficacy (57); and (3) absence of research on the precise mechanism of action of exosomes in NEC treatment. Numerous attempts have been made to overcome these limitations, such as enhancing the extraction process for exosomes and extending targeting capability by modification. Chen et al. (58) proposed an anion exchange method for efficiently extracting and detecting exosomes. Furthermore, aptamer-mediated surface modification may boost the specificity of exosomes’ ability to reach injured tissues and organs, displaying enhanced targeting capability (59–62). Exosomes’ unique properties and biological impacts must be comprehended and studied, as well as the underlying mechanism in NEC treatment and their scale-up utilizing existing technology. With sustained research, it is envisaged that exosome therapy will become one of the most promising therapies for NEC.

### Therapy with fecal microbiota transplantation

A dysbiosis of the gut microbiome is a risk factor of NEC (63). FMT, a strategy in which healthy feces are transferred to patients with dysbiosis to balance their intestinal flora, has been used to treat clostridium difficile infected diseases (64). Experimental models of NEC have shown positive results when dysbiosis is corrected with FMT. A recent study by Liu et al. concluded that FMT has a unique effect on treating NEC by decreasing inflammation in the intestines, decreasing intestinal permeability, and strengthening the intestinal barrier (65). Brunse et al. examined gut colonization patterns and host reactions to FMT according to different administration routes (66). Rectal administration is the most preferable method of administering FMT, since oral FMT administration increases lethal sepsis incidence and overall mortality by exposing the proximal gut to potentially pathogenic organisms (66). However, according to another study, intragastric administration of FMT appears safe in postsurgical newborn piglets with SBS, with no sepsis and no mortality (67). Hence, there is a need to further explore the security of administration of FMT by different routes.

### Challenge and limitation of fecal microbiota transplantation

Even though FMT has shown promising properties in preventing NEC, FMT is associated with safety concerns because no screening method will be able to exclude transfer of an infectious agent from the donor. Yan et al. suggest that the guts of recipients had higher levels of pathogenic signatures from *Escherichia coli* and *Salmonella enterica*, which may be a risk factor (68). Oral FMT administration increases lethal sepsis incidence and overall mortality by exposing the proximal gut to potentially pathogenic organisms (66). To improve the safety of FMT, Fecal filtrate transplantation (FFT) and FMT sterilization by ultraviolet radiation are techniques that remove the bacterial component from donor feces by sterile filtration (69, 70). Most studies found that fecal donors are mainly 10-day-old healthy piglets (66, 68, 69). However, there are no standard procedures for selecting donors in NEC animal models. To sum up, there are few published studies on FMT’s effects on NEC, and a greater number are still experimental. Therefore, it is essential to conduct a comprehensive screening procedure in order to determine the characteristics of FMT donors, screen conditions, the preferred route of administration and improve the quality of FMT in the future.

## Immunotherapy

### TLR4-targeting agents

Toll-like receptors (TLRs) are pattern recognition receptors (PRR) of the innate immune system, and each TLR may identify particular pathogen-associated molecular patterns (PAMP). It is generally established that TLRs have a role in NEC pathogenesis, particularly TLR4 which identifies lipopolysaccharides in Gram-negative bacteria. TLR4 was reported to be highly activated in both neonatal rats and human infants in the event of NEC (71). Researchers have shown that TLR4-deficient mice don’t display significant inflammatory responses (72, 73). Studies have demonstrated the importance of TLR4 signal activation in the development of NEC, as it can provoke excessive intestinal inflammation and increase the apoptosis and necrosis of enterocytes (74–77). TLR4-targeted agents have the potential to be useful in the treatment of NEC (Table 3).

**TABLE 3:** Targeting TLR4 by drugs in NEC.

Name	Species	Outcome	Year	References
Pregnane X receptor	Mouse	Anti-inflammation via TLR4.	2018	(72)
The secondary bile acid lithocholic acid (LCA)	Mouse	LCA activated PXR, anti-inflammation via TLR4.	2018	(72)
High mobility group box-1 inhibitor glycyrrhizin (GL)	Rat	Anti-inflammation via TLR4/NF-kB/NLRP3.	2010	(78)
Interleukin-1 (IL-1) receptor-associated kinase (IRAK) inhibitors	Rat	Anti-inflammation via TLR4.	2018	(73)

Pregnane X receptor (PXR) can function as an external biosensor and signal intermediate in producing various host-bacterial metabolites. It has been proven with an ability to inhibit TLR4 signal expression. According to an animal study, mice with PXR knockout exhibited more severe disease symptoms following experimental NEC induction (72). Lithocholic acid (LCA), a liver-distributed PXR agonist, could activate intestinal PXR, reducing NEC-related intestinal inflammation (72). The high mobility group box 1 (HMGB1) is essentially required for the incidence and progression of NEC. In animal investigations, it was revealed that when NEC developed, HMGB1 expression increased, and inflammatory cell migration was facilitated (78). Yu et al. (78) examined the effect of glycyrrhizin (GL), and HMGB1 inhibitor, in NEC and reported that it might inhibit TLR4 and the downstream NF-κB/NLRP3 signaling pathway, resulting in decreased intestinal inflammation. Hou et al. (73) revealed that an interleukin-1 receptor-associated kinase (IRAK) inhibitor lowered inflammatory factor production by downregulating TLR4 receptor expression, thereby reducing the severity of NEC-induced intestinal inflammation. The possibility that TLR4-targeted drugs particular to the pathophysiology of NEC suggest that they may represent an innovative treatment strategy.

### Challenge and limitation of immunotherapy

There is evidence that targeting TRL4 and employing biological agents to treat NEC has therapeutic effects, but related research is still in the phase of animal testing. Furthermore, the exact mechanism of action remains a mystery that must be clarified. In this context, greater emphasis should be made on the specific mode of action of TLR4-targeted drugs and appropriate biological agents to repair small intestinal injuries. As a result, it is anticipated that more effective, specialized novel drugs will be developed at the molecular level and subsequently used in NEC treatment.

## Conclusion

This review outlines lactoferrin, oligosaccharides, exosomes in breast milk, stem cells and stem cells derived-exosomes, TLR4-targeted agents, and FMT, have demonstrated promising therapeutic effects and clinical application potential for the NEC therapy. Further elucidation of mechanisms, advancements in preparation, bioengineering, and application, as well as strict clinical trials, will support the use of Lactoferrin, oligosaccharides, exosomes in breast milk, stem cells and stem cells derived-exosomes, TLR4-targeted agents, and FMT, as new therapeutics for pediatric diseases.

## Author contributions

HW, KG, and ZZ: drafting and revising manuscript. RZ, YL, QY, JL, RJ, and ZH: participating in revising the manuscript. WS and HC: reviewing the manuscript for important intellectual content. All authors read and approved the final manuscript.

## References

[1] Nino DF Sodhi CP Hackam DJ. Necrotizing enterocolitis: new insights into pathogenesis and mechanisms. *Nat Rev Gastroenterol Hepatol.* (2016) 13:590–600. 27534694 10.1038/nrgastro.2016.119 PMC5124124

[2] Thyoka M de Coppi P Eaton S Khoo K Hall NJ Curry J Advanced necrotizing enterocolitis part 1: mortality. *Eur J Pediatr Surg.* (2012) 22:8–12. 22434227 10.1055/s-0032-1306263

[3] Spencer AU Kovacevich D McKinney-Barnett M Hair D Canham J Maksym C Pediatric short-bowel syndrome: the cost of comprehensive care. *Am J Clin Nutr.* (2008) 88:1552–9. 19064515 10.3945/ajcn.2008.26007

[4] Bazacliu C Neu J. Necrotizing enterocolitis: long term complications. *Curr Pediatr Rev.* (2019) 15:115–24. 30864508 10.2174/1573396315666190312093119

[5] Weintraub AS Ferrara L Deluca L Moshier E Green RS Oakman E Antenatal antibiotic exposure in preterm infants with necrotizing enterocolitis. *J Perinatol.* (2012) 32:705–9. 22157626 10.1038/jp.2011.180

[6] Wong D Abdel-Latif M Kent A Network N. Antenatal steroid exposure and outcomes of very premature infants: a regional cohort study. *Arch Dis Child Fetal Neonatal Ed.* (2014) 99:F12–20. 24142624 10.1136/archdischild-2013-304705

[7] Downard CD Grant SN Maki AC Krupski MC Matheson PJ Bendon RW Maternal cigarette smoking and the development of necrotizing enterocolitis. *Pediatrics.* (2012) 130:78–82. 22689867 10.1542/peds.2011-3808

[8] Garcia-Munoz Rodrigo F Galan Henriquez G Figueras Aloy J Garcia-Alix Perez A. Outcomes of very-low-birth-weight infants exposed to maternal clinical chorioamnionitis: a multicentre study. *Neonatology.* (2014) 106:229–34. 10.1159/000363127 25011418

[9] Grandi C Tapia JL Cardoso VC. Impact of maternal diabetes mellitus on mortality and morbidity of very low birth weight infants: a multicenter Latin America study. *J Pediatr (Rio J).* (2015) 91:234–41. 10.1016/j.jped.2014.08.007 25433204

[10] Cetinkaya M Ozkan H Koksal N. Maternal preeclampsia is associated with increased risk of necrotizing enterocolitis in preterm infants. *Early Hum Dev.* (2012) 88:893–8. 22831636 10.1016/j.earlhumdev.2012.07.004

[11] Neu J Walker WA. Necrotizing enterocolitis. *N Engl J Med.* (2011) 364:255–64. 21247316 10.1056/NEJMra1005408 PMC3628622

[12] Denning TL Bhatia AM Kane AF Patel RM Denning PW. Pathogenesis of NEC: role of the innate and adaptive immune response. *Semin Perinatol.* (2017) 41:15–28. 27940091 10.1053/j.semperi.2016.09.014 PMC5484641

[13] Wang C Zhang M Guo H Yan J Chen L Teng W Human milk oligosaccharides activate epidermal growth factor receptor and protect against hypoxia-induced injuries in the mouse intestinal epithelium and Caco2 cells. *J Nutr.* (2020) 150:756–62. 10.1093/jn/nxz297 31915826

[14] Liu J Zhu H Li B Robinson SC Lee C O’Connell JS Lactoferrin reduces necrotizing enterocolitis severity by upregulating intestinal epithelial proliferation. *Eur J Pediatr Surg.* (2020) 30:90–5. 31344710 10.1055/s-0039-1693728

[15] Queiroz VA Assis AM Júnior Hda R. Protective effect of human lactoferrin in the gastrointestinal tract. *Rev Paul Pediatr.* (2013) 31:90–5. 23703050 10.1590/s0103-05822013000100015

[16] Nguyen DN Jiang P Stensballe A Bendixen E Sangild PT Chatterton DE. Bovine lactoferrin regulates cell survival, apoptosis and inflammation in intestinal epithelial cells and preterm pig intestine. *J Proteomics.* (2016) 139:95–102. 10.1016/j.jprot.2016.03.020 26996464

[17] Serce Pehlevan O Benzer D Gursoy T Aktas Cetin E Karatekin G Ovali MF. Cytokine responses to symbiotic and lactoferrin combination in very low birth weight neonates: a randomized control trial. *Arch Argent Pediatr.* (2020) 118:e8–15. 10.5546/aap.2020.eng.e8 31984696

[18] Manzoni P Meyer M Stolfi I Rinaldi M Cattani S Pugni L Bovine lactoferrin supplementation for prevention of necrotizing enterocolitis in very-low-birth-weight neonates: a randomized clinical trial. *Early Hum Dev.* (2014) 90:S60–5. 24709463 10.1016/S0378-3782(14)70020-9

[19] Gao Y Hou L Lu C Wang Q Pan B Wang Q Enteral lactoferrin supplementation for preventing sepsis and necrotizing enterocolitis in preterm infants: a metaanalysis with trial sequential analysis of randomized controlled trials. *Front Pharmacol.* (2020) 11:1186. 10.3389/fphar.2020.01186 32848789 PMC7426497

[20] Jantscher-Krenn E Zherebtsov M Nissan C Goth K Guner YS Naidu N The human milk oligosaccharide disialyllacto-N-tetraose prevents necrotising enterocolitis in neonatal rats. *Gut.* (2012) 61:1417–25. 22138535 10.1136/gutjnl-2011-301404 PMC3909680

[21] Autran CA Kellman BP Kim JH Asztalos E Blood AB Spence ECH Human milk oligosaccharide composition predicts risk of necrotising enterocolitis in preterm infants. *Gut.* (2018) 67:1064–70. 10.1136/gutjnl-2016-312819 28381523

[22] Zhang W He-Yang J Tu W Zhou X. Sialylated human milk oligosaccharides prevent intestinal inflammation by inhibiting toll like receptor 4/NLRP3 inflammasome pathway in necrotizing enterocolitis rats. *Nutr Metab (Lond).* (2021) 18:5. 10.1186/s12986-020-00534-z 33407596 PMC7789326

[23] Wang C Zhang M Guo H Yan J Liu F Chen J Human milk oligosaccharides protect against necrotizing enterocolitis by inhibiting intestinal damage via increasing the proliferation of crypt cells. *Mol Nutr Food Res.* (2019) 63:e1900262. 10.1002/mnfr.201900262 31207104

[24] Rasmussen SO Martin L Ostergaard MV Rudloff S Roggenbuck M Nguyen DN Human milk oligosaccharide effects on intestinal function and inflammation after preterm birth in pigs. *J Nutr Biochem.* (2017) 40:141–54. 10.1016/j.jnutbio.2016.10.011 27889684

[25] Whiteside TL. The role of tumor-derived exosomes (TEX) in shaping anti-tumor immune competence. *Cells.* (2021) 10:3054. 10.3390/cells10113054 34831276 PMC8616398

[26] Martin C Patel M Williams S Arora H Brawner K Sims B. Human breast milk-derived exosomes attenuate cell death in intestinal epithelial cells. *Innate Immun.* (2018) 24:278–84. 10.1177/1753425918785715 29991305 PMC6830917

[27] Wang X Yan X Zhang L Cai J Zhou Y Liu H Identification and peptidomic profiling of exosomes in preterm human milk: insights into necrotizing enterocolitis prevention. *Mol Nutr Food Res.* (2019):e1801247. 10.1002/mnfr.201801247 31067344

[28] Dong P Zhang Y Yan DY Wang Y Xu X Zhao YC Protective effects of human milk-derived exosomes on intestinal stem cells damaged by oxidative stress. *Cell Transplant.* (2020) 29:963689720912690. 10.1177/0963689720912690 32193954 PMC7444213

[29] Gao R Zhang R Qian T Peng X He W Zheng S A comparison of exosomes derived from different periods breast milk on protecting against intestinal organoid injury. *Pediatr Surg Int.* (2019) 35:1363–8. 10.1007/s00383-019-04562-6 31576466

[30] Hock A Miyake H Li B Lee C Ermini L Koike Y Breast milk-derived exosomes promote intestinal epithelial cell growth. *J Pediatr Surg.* (2017) 52:755–9. 28188035 10.1016/j.jpedsurg.2017.01.032

[31] Xie MY Hou LJ Sun JJ Zeng B Xi QY Luo JY Porcine milk exosome MiRNAs attenuate LPS-induced apoptosis through inhibiting TLR4/NF-kappaB and p53 pathways in intestinal epithelial cells. *J Agric Food Chem.* (2019) 67:9477–91. 10.1021/acs.jafc.9b02925 31429552

[32] Li B Hock A Wu RY Minich A Botts SR Lee C Bovine milk-derived exosomes enhance goblet cell activity and prevent the development of experimental necrotizing enterocolitis. *PLoS One.* (2019) 14:e0211431. 10.1371/journal.pone.0211431 30699187 PMC6353182

[33] Shao A Tu S Lu J Zhang J. Crosstalk between stem cell and spinal cord injury: pathophysiology and treatment strategies. *Stem Cell Res Ther.* (2019) 10:238. 10.1186/s13287-019-1357-z 31387621 PMC6683526

[34] Stonesifer C Corey S Ghanekar S Diamandis Z Acosta SA Borlongan CV. Stem cell therapy for abrogating stroke-induced neuroinflammation and relevant secondary cell death mechanisms. *Prog Neurobiol.* (2017) 158:94–131. 10.1016/j.pneurobio.2017.07.004 28743464 PMC5671910

[35] Tayman CN Uckan D Ulus AT Tonbul A Hirfanoglu IM Helvacioglu F mesenchymal stem cell therapy in necrotizing enterocolitis a rat study. *Pediatr Res.* (2011) 70:489–94. 21772224 10.1203/PDR.0b013e31822d7ef2

[36] McCulloh CJ Olson JK Wang Y Zhou Y Tengberg NH Deshpande S Treatment of experimental necrotizing enterocolitis with stem cell-derived exosomes. *J Pediatr Surg.* (2018) 53:1215–20. 29661576 10.1016/j.jpedsurg.2018.02.086 PMC5994352

[37] Yang J Watkins D Chen C Bhushan B Zhou Y Besner G. Heparin-binding epidermal growth factor-like growth factor and mesenchymal stem cells act synergistically to prevent experimental necrotizing enterocolitis. *J Am Coll Surg.* (2012) 215:534–45. 10.1016/j.jamcollsurg.2012.05.037 22819639 PMC3444529

[38] Murphy SV Atala A. Amniotic fluid and placental membranes: unexpected sources of highly multipotent cells. *Semin Reprod Med.* (2013) 31:62–8. 10.1055/s-0032-1331799 23329638

[39] Bottai D Cigognini D Nicora E Moro M Grimoldi MG Adami R Third trimester amniotic fluid cells with the capacity to develop neural phenotypes and with heterogeneity among sub-populations. *Restor Neurol Neurosci.* (2012) 30:55–68. 10.3233/RNN-2011-0620 22377907

[40] McCulloh CJ Olson JK Zhou Y Wang Y Besner GE. Stem cells and necrotizing enterocolitis: a direct comparison of the efficacy of multiple types of stem cells. *J Pediatr Surg.* (2017) 52:999–1005. 10.1016/j.jpedsurg.2017.03.028 28366560 PMC5467690

[41] McCulloh CJ Olson JK Wang Y Vu J Gartner S Besner GE. Evaluating the efficacy of different types of stem cells in preserving gut barrier function in necrotizing enterocolitis. *J Surg Res.* (2017) 214:278–85. 10.1016/j.jss.2017.03.026 28624056 PMC5474934

[42] Pisano C Besner GE. Potential role of stem cells in disease prevention based on a murine model of experimental necrotizing enterocolitis. *J Pediatr Surg.* (2019) 54:413–6. 10.1016/j.jpedsurg.2018.07.025 30236604 PMC6380911

[43] Li B Lee C O’Connell JS Antounians L Ganji N Alganabi M Activation of Wnt signaling by amniotic fluid stem cell-derived extracellular vesicles attenuates intestinal injury in experimental necrotizing enterocolitis. *Cell Death Dis.* (2020) 11:750. 10.1038/s41419-020-02964-2 32929076 PMC7490270

[44] Kagia A Tzetis M Kanavakis E Perrea D Sfougataki I Mertzanian A Therapeutic effects of mesenchymal stem cells derived from bone marrow, umbilical cord blood, and pluripotent stem cells in a mouse model of chemically induced inflammatory bowel disease. *Inflammation.* (2019) 42:1730–40. 10.1007/s10753-019-01033-x 31227956

[45] Drucker NA Te Winkel JP Shelley WC Olson KR Markel TA. Inhibiting hydrogen sulfide production in umbilical stem cells reduces their protective effects during experimental necrotizing enterocolitis. *J Pediatr Surg.* (2019) 54:1168–73. 10.1016/j.jpedsurg.2019.02.037 30879750 PMC6545254

[46] Rager TM Olson JK Zhou Y Wang Y Besner GE. Exosomes secreted from bone marrow-derived mesenchymal stem cells protect the intestines from experimental necrotizing enterocolitis. *J Pediatr Surg.* (2016) 51:942–7. 10.1016/j.jpedsurg.2016.02.061 27015901 PMC4921266

[47] Villamor-Martinez E Hundscheid T Kramer BW Hooijmans CR Villamor E. Stem cells as therapy for necrotizing enterocolitis: a systematic review and meta-analysis of preclinical studies. *Front Pediatr.* (2020) 8:578984. 10.3389/fped.2020.578984 33363060 PMC7755993

[48] Akduman H Dilli D Ergun E Cakmakci E Celebi SK Citli R Successful mesenchymal stem cell application in supraventricular tachycardia-related necrotizing enterocolitis: a case report. *Fetal Pediatr Pathol.* (2019) 40:250–55. 10.1080/15513815.2019.1693672 31755792

[49] Schwartz SD Hubschman J-P Heilwell G Franco-Cardenas V Pan CK Ostrick RM Embryonic stem cell trials for macular degeneration: a preliminary report. *Lancet.* (2012) 379:713–20. 22281388 10.1016/S0140-6736(12)60028-2

[50] Moradi S Mahdizadeh H Saric T Kim J Harati J Shahsavarani H Research and therapy with induced pluripotent stem cells (iPSCs): social, legal, and ethical considerations. *Stem Cell Res Ther.* (2019) 10:341. 10.1186/s13287-019-1455-y 31753034 PMC6873767

[51] Deng J Zhang Y Xie Y Zhang L Tang P. Cell transplantation for spinal cord injury: tumorigenicity of induced pluripotent stem cell-derived neural stem/progenitor cells. *Stem Cells Int.* (2018) 2018:5653787. 10.1155/2018/5653787 PMC5817265 29535771

[52] Kalra H Adda CG Liem M Ang CS Mechler A Simpson RJ Comparative proteomics evaluation of plasma exosome isolation techniques and assessment of the stability of exosomes in normal human blood plasma. *Proteomics.* (2013) 13:3354–64. 10.1002/pmic.201300282 24115447

[53] Liga A Vliegenthart AD Oosthuyzen W Dear JW Kersaudy-Kerhoas M. Exosome isolation: a microfluidic road-map. *Lab Chip.* (2015) 15:2388–94. 10.1039/c5lc00240k 25940789

[54] Tauro BJ Greening DW Mathias RA Ji H Mathivanan S Scott AM Comparison of ultracentrifugation, density gradient separation, and immunoaffinity capture methods for isolating human colon cancer cell line LIM1863-derived exosomes. *Methods.* (2012) 56:293–304. 10.1016/j.ymeth.2012.01.002 22285593

[55] Wan Y Cheng G Liu X Hao SJ Nisic M Zhu CD Rapid magnetic isolation of extracellular vesicles via lipid-based nanoprobes. *Nat Biomed Eng.* (2017) 1:0058. 10.1038/s41551-017-0058 28966872 PMC5618714

[56] Woo HK Sunkara V Park J Kim TH Han JR Kim CJ Exodisc for rapid, size-selective, and efficient isolation and analysis of nanoscale extracellular vesicles from biological samples. *ACS Nano.* (2017) 11:1360–70. 10.1021/acsnano.6b06131 28068467

[57] Fuhrmann G Chandrawati R Parmar PA Keane TJ Maynard SA Bertazzo S Engineering extracellular vesicles with the tools of enzyme prodrug therapy. *Adv Mater.* (2018) 30:e1706616. 10.1002/adma.201706616 29473230 PMC5901706

[58] Chen J Xu Y Lu Y Xing W. Isolation and visible detection of tumor-derived exosomes from plasma. *Anal Chem.* (2018) 90:14207–15. 10.1021/acs.analchem.8b03031 30372048

[59] Liu C Zhao J Tian F Chang J Zhang W Sun J. lambda-DNA- and aptamer-mediated sorting and analysis of extracellular vesicles. *J Am Chem Soc.* (2019) 141:3817–21. 10.1021/jacs.9b00007 30789261

[60] Luo ZW Li FX Liu YW Rao SS Yin H Huang J Aptamer-functionalized exosomes from bone marrow stromal cells target bone to promote bone regeneration. *Nanoscale.* (2019) 11:20884–92. 10.1039/c9nr02791b 31660556

[61] Tran PH Xiang D Nguyen TN Tran TT Chen Q Yin W Aptamer-guided extracellular vesicle theranostics in oncology. *Theranostics.* (2020) 10:3849–66. 10.7150/thno.39706 32226524 PMC7086349

[62] Zou J Shi M Liu X Jin C Xing X Qiu L Aptamer-functionalized exosomes: elucidating the cellular uptake mechanism and the potential for cancer-targeted chemotherapy. *Anal Chem.* (2019) 91:2425–30. 10.1021/acs.analchem.8b05204 30620179 PMC6662586

[63] Brehin C Dubois D Dicky O Breinig S Oswald E Serino M. Evolution of gut microbiome and metabolome in suspected necrotizing enterocolitis: a case-control study. *J Clin Med.* (2020) 9:2278. 10.3390/jcm9072278 32709038 PMC7408695

[64] Kelly CR Khoruts A Staley C Sadowsky MJ Abd M Alani M Effect of fecal microbiota transplantation on recurrence in multiply recurrent clostridium difficile infection: a randomized trial. *Ann Intern Med.* (2016) 165:609–16. 10.7326/M16-0271 27547925 PMC5909820

[65] Liu J Miyake H Zhu H Li B Alganabi M Lee C Fecal microbiota transplantation by enema reduces intestinal injury in experimental necrotizing enterocolitis. *J Pediatr Surg.* (2020) 55:1094–8. 10.1016/j.jpedsurg.2020.02.035 32234317

[66] Brunse A Martin L Rasmussen TS Christensen L Skovsted Cilieborg M Wiese M Effect of fecal microbiota transplantation route of administration on gut colonization and host response in preterm pigs. *ISME J.* (2019) 13:720–33. 10.1038/s41396-018-0301-z 30367124 PMC6461782

[67] Hinchliffe T Pauline ML Wizzard PR Jovel J Nation PN Wales PW The effect of fecal microbial transplant on intestinal microbial composition in short-bowel neonatal piglets. *JPEN J Parenter Enteral Nutr.* (2022) 10.1002/jpen.2333 35043436

[68] Hui Y Vestergaard G Deng L Kot WP Thymann T Brunse A Donor-dependent fecal microbiota transplantation efficacy against necrotizing enterocolitis in preterm pigs. *NPJ Biofilms Microbiomes.* (2022) 8:48. 10.1038/s41522-022-00310-2 35680942 PMC9184500

[69] Brunse A Deng L Pan X Hui Y Castro-Mejia JL Kot W Fecal filtrate transplantation protects against necrotizing enterocolitis. *ISME J.* (2022) 16:686–94. 10.1038/s41396-021-01107-5 34552194 PMC8857206

[70] Prado C Abatti MR Michels M Corneo E Cucker L Borges H Comparative effects of fresh and sterile fecal microbiota transplantation in an experimental animal model of necrotizing enterocolitis. *J Pediatr Surg.* (2022) 10.1016/j.jpedsurg.2021.12.013 35058059

[71] Liu T Zong H Chen X Li S Liu Z Cui X Toll-like receptor 4-mediated necroptosis in the development of necrotizing enterocolitis. *Pediatr Res.* (2021) 91:73–82. 10.1038/s41390-021-01457-y 33731807 PMC8770135

[72] Huang K Mukherjee S DesMarais V Albanese JM Rafti E Draghi Ii A Targeting the PXR-TLR4 signaling pathway to reduce intestinal inflammation in an experimental model of necrotizing enterocolitis. *Pediatr Res.* (2018) 83:1031–40. 10.1038/pr.2018.14 29360809 PMC5959752

[73] Hou Y Lu X Zhang Y. IRAK Inhibitor protects the intestinal tract of necrotizing enterocolitis by inhibiting the toll-like receptor (TLR) inflammatory signaling pathway in rats. *Med Sci Monit.* (2018) 24:3366–73. 10.12659/MSM.910327 29784900 PMC5992962

[74] Jia H Sodhi CP Yamaguchi Y Lu P Ladd MR Werts A Toll like receptor 4 mediated lymphocyte imbalance induces nec-induced lung injury. *Shock.* (2019) 52:215–23. 10.1097/SHK.0000000000001255 30148762 PMC6387863

[75] Du M Yuan L Tan X Huang D Wang X Zheng Z The LPS-inducible lncRNA Mirt2 is a negative regulator of inflammation. *Nat Commun.* (2017) 8:2049. 10.1038/s41467-017-02229-1 29230038 PMC5725456

[76] Egan CE Sodhi CP Good M Lin J Jia H Yamaguchi Y Toll-like receptor 4-mediated lymphocyte influx induces neonatal necrotizing enterocolitis. *J Clin Invest.* (2016) 126:495–508. 26690704 10.1172/JCI83356 PMC4731173

[77] Zhou Y Li Y Zhou B Chen K Lyv Z Huang D Inflammation and apoptosis: dual mediator role for toll-like receptor 4 in the development of necrotizing enterocolitis. *Inflamm Bowel Dis.* (2017) 23:44–56. 10.1097/MIB.0000000000000961 27849634

[78] Dai S Sodhi C Cetin S Richardson W Branca M Neal MD Extracellular high mobility group box-1 (HMGB1) inhibits enterocyte migration via activation of toll-like receptor-4 and increased cell-matrix adhesiveness. *J Biol Chem.* (2010) 285:4995–5002. 10.1074/jbc.M109.067454 20007974 PMC2836103

